# Beyond the Numbers: Exploring Tensions Between Formal Entrustment and Trainee Readiness in Internship Training — A Mixed-Methods Study

**DOI:** 10.5334/pme.2259

**Published:** 2026-03-19

**Authors:** Tarik Al-Diery, Sally Marotti, Yu Ting Sim, Myriam Jaam, Debra Rowett, Jacinta L. Johnson

**Affiliations:** 1 Clinical Pharmacy and Practice, College of Pharmacy, QU Health, Qatar University, Doha, Qatar; 2 School of Pharmacy and Biomedical Sciences, College of Health, Adelaide University, Adelaide, South Australia, Australia; 3 SA Pharmacy, SA Health, South Australia, Australia; 4 Digital Health Research Lab, Flinders Health and Medical Research Institute, Flinders University, Bedford Park, SA, Australia; 5 College of Pharmacy, QU Health, Qatar University, Qatar

## Abstract

**Purpose:**

Entrustment decision-making is shaped by both supervisor and trainee. Existing research has focused largely on supervisor decision-making; however, less is known about how trainees experience and internalize entrustment. This study explores how trainee entrustment develops over a pharmacy internship and examines lived experiences that influence progression toward unsupervised practice.

**Methods:**

A convergent mixed-methods design explored the entrustment journey of provisionally registered (intern) pharmacists in Australia. Quantitative data were collected via self-administered questionnaires at three time points (beginning, middle, end of internship), capturing perceptions of entrustable professional activities (EPA) utility and self-perceived readiness for entrustment across ten EPAs. At year-end, focus groups explored pharmacy interns’ perceptions of the EPA framework and how they interpreted and internalized it in relation to their development. Qualitative data were analyzed using reflexive thematic analysis and triangulated with quantitative findings to examine convergence and divergence over time.

**Results:**

Seventeen pharmacy interns completed questionnaires; 16 of these participated in focus groups. Self-perceived readiness for entrustment increased significantly across most EPAs, with median entrustment ratings reaching level 3 (independent with reactive supervision) by the end of internship. Pharmacy interns described that being entrusted supported confidence development, particularly when paired with structured feedback and reflective practice. Some noted that an emphasis on meeting numeric benchmarks risked reducing the process to a “numbers game,” overshadowing diverse and complex learning opportunities, and diminishing feedback opportunities once level 3 was achieved. At times, this created asymmetry between being entrusted by a supervisor and the interns’ own sense of readiness for more complex practice.

**Conclusion:**

EPAs support trainees’ progression toward autonomy by embedding structure, feedback, and self-reflection that affirms competence and guides development. When entrustment decisions are tied to achieving a predetermined number of EPA observations, there is a risk that entrustment may be conferred prematurely, thereby reducing the depth of feedback and reflection that follows. When framed as assessments *for* learning, EPAs better support trainees’ transition to confident, autonomous practice.

## Introduction

The transition from supervised trainee to autonomous practitioner marks a critical stage in trainee development, encompassing not only the demonstration of technical competence but also the readiness to manage uncertainty, seek feedback, and take accountability for clinical decisions [1,2,3,4]. The journey to unsupervised practice requires a deliberate and calibrated progression of responsibility, where supervisors navigate an intricate balance of granting trainees increasing autonomy while ensuring patient safety and high standards of care [5,6,7,8,9,10,11]. The concept of entrustment is central to this developmental process, where supervisors make informed context-sensitive decisions to afford trainees with greater autonomy to demonstrate both technical competence and general trustworthiness as they perform specific profession-based activities [12,13,14,15,16,17,18].

Entrustable professional activities (EPAs) have emerged as a transformative framework for competency-based education, with entrustment as an outcome for trainee readiness for unsupervised practice [2,6,19,20]. Entrustment decision-making is a complex psychosocial contextual process that extends beyond the trainees’ competence and trustworthiness [3,15,20,21,22,23]. Supervisors’ trust-propensity, contextual settings, perceived task complexity, and the evolving supervisor-trainee relationship are factors that influence the dynamics of how supervisors make entrustment decisions [17,18,24,25,26,27,28,29,30]. While entrustment decision-making has expanded within workplace-based training settings, questions remain regarding how entrustment unfolds in real-world practice and the extent to which formal assessments of entrustment capture trainees’ lived experiences of graded autonomy, developmental needs, and professional identity formation [24,31,32].

A growing tension has emerged between the use of entrustment scales for assessment of outcomes and their intended role in supporting trainee development [31,32,33]. While numeric ratings offer a structured way to communicate the level of supervision required, they often fall short in capturing the nuanced reasoning behind an entrustment decision or generating meaningful feedback to guide trainee growth [34,35,36]. Without meaningful structured feedback, these numeric ratings risk becoming decontextualized judgments, devoid of the coaching function needed to support the trainee in reflective learning [34,37]. Guided self-reflection and constructive narrative feedback plays a critical role in adding interpretive depth to numeric ratings, bridging the gap between judgment and justification [34,38]. Yet, a persistent challenge lies in the dual function of entrustment ratings: they are required to support high-stakes decisions while simultaneously fostering trainee development [32,34,35]. In practice, however, these ratings are often used primarily to justify entrustment encounters, leaving limited room for formative guidance or meaningful feedback [32,34,35,39]. Research in medical education has shown that trainees perceive greater value in the verbal feedback and reflective discussions that accompany formative entrustment observations than in the numeric entrustment ratings themselves [31,32,33]. When formative entrustment observations are reduced strictly to an assessment exercise, they risk undermining opportunities for meaningful feedback and deep learning [31,32,33,36].

Existing literature has predominately examined entrustment from the supervisor’s perspective, focusing on decision-making frameworks [17,22] and entrustment assessment reliability [40,41,42,43,44]. While valuable, this approach provides limited insight into how trainees experience and internalize the entrustment process [24,31,32,33]. Moreover, our understanding of how trainees themselves experience entrustment across the continuum of training remains limited [24,33]. For trainees, being entrusted is not merely about being deemed competent; it represents a shift in identity, accountability, and recognition as a collaborative healthcare professional committed to the practice of lifelong learning [24]. The affective dimensions of entrustment and how it is experienced, interpreted, and internalized play a critical role in shaping a trainee’s confidence, motivation, and readiness for unsupervised practice [31,32,33].

Despite growing interest in entrustment as a developmental milestone and assessment outcome, little is known about how trainees themselves experience this progression over time, or how contextual factors shape their path toward autonomy. This study aims to explore the longitudinal development of trainee entrustment across a pharmacy internship, and to understand the perceptions, lived experiences, and contextual factors that shape a trainee’s journey toward unsupervised practice.

## Methods

### Study design

This mixed-methods study explored the longitudinal trajectories of entrustment and pharmacy trainees’ progression toward unsupervised practice. A convergent design was used to collect quantitative data on trainees’ self-perceived readiness for entrustment at three time points (beginning, middle, and end of internship), followed by qualitative data via focus group interviews capturing attitudes, perceptions, and lived experiences with EPAs and the entrustment process at the completion of training.

A mixed-methods approach was essential because entrustment represents both a measurable construct (assessed through validated entrustment scales) and a complex psychosocial process [45]. Quantitative data alone cannot capture the complex relationship between entrustment and trainee development. Qualitative methods can provide deeper insights into trainee perceptions of EPAs across diverse settings. The integration of both quantitative and qualitative data through triangulation enabled a more comprehensive understanding of how entrustment evolves during internship training, revealing points of convergence as well as divergence that would not have been apparent through either method alone. The reporting of this study was guided by the Good Reporting of a Mixed Methods Study (GRAMMS) checklist (Appendix A), ensuring transparent and comprehensive presentation of methods and findings.

### Study setting and participants

The study was conducted with provisionally registered (intern) pharmacists completing their training within SA Pharmacy, the South Australian statewide public hospital pharmacy service, which services a network of metropolitan and regional hospitals. In Australia, students are required to complete a pharmacy degree program to be eligible for registration with the Pharmacy Board of Australia, before undertaking 1575 hours of supervised practice as an intern [46,47]. Interns within SA Pharmacy rotate through various clinical and operational settings to consolidate knowledge and skills.

Intern performance is assessed against ten locally defined EPAs developed by SA Pharmacy (Appendix B), of which six are designated as core EPAs and four are optional, and are assessed only where they are applicable and observable within the intern’s practice setting. The core EPAs represent routine clinical and operational activities that interns are expected to perform repeatedly across most rotations (i.e. daily to multiple times per week), whereas the optional EPAs reflect more specialized tasks that are encountered only in specific clinical or operational contexts. Interns are expected to complete a minimum of ten documented observations at entrustment level 3 (independent with reactive supervision) for each applicable core EPA. Drawing on Olle ten Cate’s original five-level entrustment scale, SA Pharmacy adapted the five-level scale to include a sublevel within level 2 for a more granular supervision assessment (Table 1).

**Table 1:** The entrustment scale used at South Australia Health in the intern training year.


LEVEL OF ENTRUSTMENT	

Level 1	The learner is able to thoughtfully observe

Level 2a	The learner is able to perform the task with regular direct supervision and proactive guidance

Level 2b	The learner is able to perform the task with occasional direct supervision and proactive guidance

Level 3	The learner is able to perform the tasks independently with reactive supervision

Level 4	The learner can perform the task in complex populations and environments

Level 5	The learner is able to supervise more junior colleagues


All 21 interns undertaking the 2024 program were invited to participate. Participants received a participant information form and provided written consent. Non-responders were followed up after two weeks with no further recruitment attempts.

### Phase 1: Quantitative data collection of self-perceived readiness for entrustment to perform SA Pharmacy EPAs and the utility of EPAs

An anonymous online self-administered questionnaire exploring intern pharmacists’ self-perceived readiness for entrustment to perform different EPAs and their attitudes and perceptions toward EPAs was adapted from earlier entrustment research by members of the research team [13] and Pittenger and colleagues [48]. In this study, self-perceived readiness for entrustment referred to interns’ self-assessment of the level of supervision they believed they required for each EPA, mapped onto the institutional entrustment scale. The questionnaire was administered in early April, early August, and late November 2024, corresponding to the beginning (January–March), middle (April–July), and end (August–November) of the internship year. Questions explored interns’ confidence in using EPAs and assessed whether the EPA framework supported self-reflection, guided the completion of workplace-based activities, and helped identify knowledge and skill gaps. The baseline questionnaire administered at the first time point also collected demographic data. All questionnaires were administered through Research Electronic Data Capture (REDCap), a secure electronic data capture platform hosted at the University of South Australia [49,50].

Due to the ordinal nature of the Likert scale data, non-parametric statistical tests were used to analyze changes in interns’ self-perceived readiness for entrustment and attitudes toward EPAs across the three time points. Since individual participant responses were anonymous and were not matched across time points, data were treated as independent samples and analyzed using the Kruskal-Wallis test to determine significant differences in self-perceived readiness for entrustment ratings between time points. Following significant Kruskal-Wallis results, post-hoc pairwise comparisons were conducted using Dunn’s test with Bonferroni correction to control for Type I error across multiple comparisons. The alpha level was set at .05 for all analyses. Statistical analyses were performed using IBM SPSS Statistics version 30.

### Phase 2: Focus group interviews

Interns who consented to participate in the surveys were invited to participate in focus group interviews between October and November 2024. The participant information sheet and an online consent form were distributed via REDCap [49,50]. The qualitative component of this study aimed to enrich and complement the questionnaire findings by capturing how trainees made sense of entrustment experiences over time and the utility of EPAs in supporting their development to unsupervised practice.

An interview guide was developed to explore participants’ perspectives on entrustment, EPA use, and learning trajectories. To support triangulation with the survey, the guide was informed by Zimmerman’s self-regulated learning framework, which conceptualizes how learners plan and set goals, self-monitor, self-reflect, and self-evaluate their development [51]. The interview guide was piloted with two former SA Pharmacy interns with prior experience using EPAs and the entrustment framework, and the resulting feedback was used to refine the wording and structure of the guide to ensure clarity and alignment with the study objectives.

Five online focus group interviews were conducted in November 2024 via Microsoft Teams (Microsoft Corporation, Redmond, Washington, USA), with each session scheduled for approximately 60 minutes. Participants who provided informed consent were assigned to focus groups based on availability. All interviews were conducted by the lead investigator (TD), with the co-investigator (JJ) serving as moderator and primary note-taker. During each session, the lead investigator presented interview questions via PowerPoint slides to facilitate engagement and clarity. An iterative approach to data collection was adopted, whereby emerging insights from earlier focus groups informed subsequent discussions to ensure a comprehensive exploration of the intern experience with entrustment.

All interviews were audio-recorded and transcribed verbatim by the lead investigator using the transcription software Otter.ai. Transcripts were anonymized using unique participant codes to maintain confidentiality. A reflexive thematic analysis was conducted following the six-phase approach outlined by Braun and Clarke [52]. An inductive approach was chosen to generate themes related to trainees’ lived experiences with entrustment and the utility of the EPA framework. Two members of the research team (TD and YS), both experienced in qualitative methods and entrustment research, independently coded the transcripts using Microsoft Word (Microsoft Corporation, Redmond, Washington, USA). Each investigator assigned descriptive labels to specific data segments (words, sentences, or phrases) that reflected key ideas related to entrustment decision-making and EPA use.

Following independent coding, the researchers met regularly to compare interpretations, reconcile differences, and collaboratively develop a coding framework. This framework was iteratively refined through discussion until a shared understanding of the analytic interpretations was achieved, with codes subsequently organized into subthemes and overarching themes. Representative quotes from participants were selected to illustrate each theme. These themes were examined alongside the quantitative findings to highlight areas of convergence and divergence.

To ensure the credibility and trustworthiness of the analysis, the research team followed Lincoln and Guba’s criteria for rigor, incorporating investigator triangulation, reflexive auditing, and group peer debriefing [53]. Throughout the data collection and analysis phases, investigators (TD, YS, SM and JJ), all with experience as pharmacy educators and clinical supervisors, maintained a reflexivity journal to document reflections on their assumptions, positionality, and potential influence on data collection and interpretation.

### Triangulation of results

In keeping with the convergent mixed-methods design, quantitative and qualitative findings were integrated through triangulation to enhance the depth and validity of interpretation. Self-perceived readiness for entrustment ratings and attitudes toward EPA utility collected at three time points were systematically compared with themes emerging from focus group interviews. This analytic integration allowed us to identify areas of convergence, where quantitative patterns aligned with interns’ reported experiences, as well as divergence, where numeric trends contrasted with perceptions of readiness or EPA utility. Through this process, quantitative results were contextualized and enriched by interns’ lived experiences, providing a comprehensive account of how entrustment was understood and internalized across the internship year. As the focus of this study was to explore the lived experiences of pharmacy interns with respect to entrustment and EPA utility, a greater emphasis was placed on the qualitative phase of this study.

## Results

### Phase 1: Quantitative questionnaire

Seventeen of the 21 SA Pharmacy interns participated in the longitudinal study, completing the self-perceived readiness for entrustment and EPA utility questionnaire at three time points. The mean participant age was 25.2 ± 6.5 years. Twelve interns reported more than 500 hours of prior pharmacy experience beyond their university placements, and all were familiar with EPAs before starting internship.

Interns’ self-perceived readiness for entrustment ratings increased significantly for five of the six core EPAs from the beginning to the end of internship. By year-end, the median entrustment level for all core EPAs reached level 3 (Table 2). The greatest change occurred between time point 1 (January–March) and time point 2 (April–July), except for EPA 4 (therapeutic drug monitoring), where improvement was evident between time points 2 and 3 (August–November). Notably, perceived entrustment for EPA 1 (dispensing) declined from a median entrustment of level 4 at time point 2 to level 3 at time point 3.

**Table 2:** Intern pharmacists’ (n = 17) self-reported entrustment ratings at performing different entrustable professional activities across three time points in the intern training year and the corresponding changes in self-reported readiness for entrustment.


ENTRUSTABLE PROFESSIONAL ACTIVITY	MEDIAN SELF-REPORTED READINESS FOR ENTRUSTMENT T1*: JAN–MAR	MEDIAN SELF-REPORTED READINESS FOR ENTRUSTMENT T2**: APR–JUL	MEDIAN SELF-REPORTED READINESS FOR ENTRUSTMENT T3***: AUG–NOV	PAIRWISE COMPARISON OF T1 TO T2	PAIRWISE COMPARISON OF T1 TO T3	PAIRWISE COMPARISON OF T2 TO T3	OVERALL COMPARISON OF THE THREE TIME POINTS (T1, T2, AND T3)

				*p* VALUE°	*p* VALUE°	*p* VALUE°	*p* VALUE‡

EPA1: Dispensing medications	3	4	3	.473	.492	.163	.505

EPA 2: Taking a medication history	3	3	3	**.024**	**.024**	1	**.031**

EPA 3: Conducting a chart annotation, clinical review, and assessment of medication management	2b	3	3	**.016**	**.070**	.287	**.001**

EPA 4: Conducting therapeutic drug monitoring	2b	2b	3	**.003**	**.000**	.310	**.001**

EPA 5: Facilitating patient discharge or transfer	2b	3	3	**.015**	**.015**	.219	**.001**

EPA 6: Providing specific patient education on inhaler technique, anticoagulation counselling, oral cancer therapy education etc)	2b	3	3	**.013**	**.001**	.287	**.001**

EPA 7: Initiation of clozapine	2a	2a	2b	**.021**	**.001**	.169	**.024**

EPA 8: Participating in an interprofessional ward round	2a	2b	2b	**.001**	**.001**	**.024**	**.021**

EPA 9: Providing regional cancer services – related tasks (i.e ordering and receiving cancer therapies)	1	1	1	.132	.132	1	.182

EPA 10: Conducting Partner Pharmacist Medication Charting (PPMC)	1	1	1	.545	**.034**	.106	.057

*T1 = Time point 1							

**T2 = Time point 2							

***T3 = Time point 3							


° *p* value from the Dunn test with the Bonferroni adjustment comparing changes in intern pharmacists’ self-reported median entrustment to perform different entrustable professional activities between each of the three different time points. Bolded results are statistically significant differences between the different groups with a *p* value of < 0.05. ‡ *p* value from the Kurskal-Wallis test comparing changes in intern pharmacists’ self-reported median entrustment to perform different entrustable professional activities across the three different time points. Bolded results are statistically significant differences between the different groups with a *p* value of < 0.05.

For the optional EPAs, significant improvement was noted only for EPA 7 (clozapine initiation) and EPA 8 (interprofessional ward rounds). By the end of the internship, the median self-assigned entrustment level for EPAs 7 and 8 remained at 2b, suggesting trainees still felt they required occasional direct supervision. No change was reported for the EPAs 9 and 10 which remained at level 1, with trainees noting they perceived they were only capable of observing for these EPAs.

**Figure 1:**
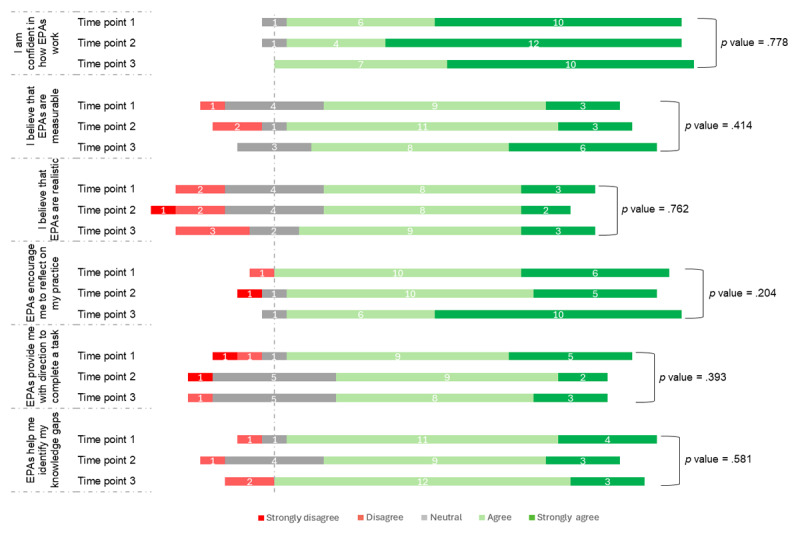
Longitudinal changes in intern pharmacists’ (n = 17) attitudes and perceptions of Entrustable Professional Activities across the pharmacy internship.

Interns’ perceptions of EPA utility appeared largely stable across the year (Figure 1). There was a trend toward increased agreement that EPAs encouraged self-reflection between time points two and three, while perceptions of EPAs being realistic and helping identify knowledge gaps showed a slight decline over the same period.

### Phase 2: Qualitative interviews

**Figure 2:**
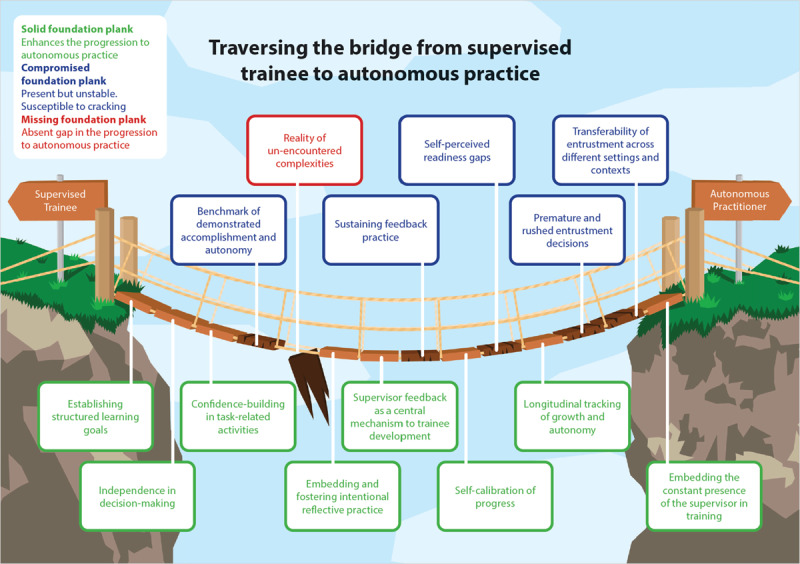
Visual synthesis of the thematic analysis depicting trainees’ perceived journey toward unsupervised practice, represented as a wooden bridge with each subtheme illustrated as an individual wooden plank.

Sixteen of the 17 survey respondents participated in one of five focus group interviews, with sessions averaging 57.5 minutes. Reflexive thematic analysis generated four major themes describing interns’ lived experiences with entrustment: (1) Entrustment as a time and task-dependent marker of competence, readiness, and autonomy, with EPAs providing structured learning and goal setting; (2) Feedback and self-reflection as drivers of trainee development; (3) Mismatch between formal entrustment and the trainees’ self-perceived readiness; and (4) Perceptions and implications upon reaching a finite numerical target leading to autonomy in practice. Table 3 provides a summary of the themes, subthemes, and representative quotes. Figure 2 visualizes the subthemes as planks on a wooden suspension bridge, coming together to represent a path facilitating the intern’s journey to autonomous practice.

**Table 3:** Themes, contextual insights, and representative quotes illustrating intern pharmacists’ experiences of entrustment progression across the internship year


THEME	SUBTHEME AND CONTEXT	DESCRIPTION OF CONTEXT	REPRESENTATIVE QUOTES

Entrustment as a time and task-dependent marker of competence, readiness, and autonomy, with EPAs providing structured learning and goal setting	Confidence-building in task-related activities	As trainees achieve successive levels of entrustment, they internalize trust from supervisors as confidence in their own abilities, promoting readiness to manage tasks with greater autonomy	“For me, a big takeaway from having done all of my EPAs is now having the confidence to figure out a plan of what to do if I’m faced with a task where I’m not too sure.” – Intern 5

	Independence in decision-making	Autonomy through accountable decision-making within a collaborative team context	“You’re still working within a team, and you’re still getting help from other people. But I think that’s where the independent kind of phrase comes in, independent in decision making.” – Intern 10

	Benchmark of demonstrated accomplishment and autonomy	Entrustment through EPAs were regarded as departmental markers of practice readiness	“Essentially, it was kind of viewed that once you had completed an EPA ten times at level 3, pharmacists were more than happy to kind of, trust you to go and perform those activities without supervision.” – Intern 3

	Establishing structured learning goals	EPAs helped establish clear learning goals for the trainees and the expectations throughout training	“I feel like EPAs were very useful for our development. They gave us an aim to work towards, so we knew what we were doing and what we were aiming for, and that really helped us set goals for ourselves throughout the year.” – Intern 12 “It’s a learning tool, and the tool that’s being used to show, I guess, your skills and to develop them to the point where you are trusted to do the activity independently.” – Intern 4

	Longitudinal tracking of growth and autonomy	EPAs allowed both trainees and supervisors to monitor the growth of the trainee throughout the intern year	“An EPA over time is like a journey that records our improvement to the point where an employer can say, ‘yes, you can do these tasks independently,’ and it provides a record of that progression.” – Intern 14 “That’s not necessarily measured every time, but it starts to reflect that you are being more self-directed, and that you can be more independent in doing different activities.” – Intern 2

	Embedding the constant presence of the supervisor in training	The structure of entrustment through observation from a supervisor ensured trainees were supported by the presence of a supervisor	“In terms of development, it is a good tool to ensure that a pharmacist is with you so you’re not practicing on your own.” – Intern 6 “The EPA system almost ensured that we had a supervisor with us to support us at different stages of training….I know that’s not always the case but having that almost mandated because of the system was beneficial to my development.” – Intern 8

Feedback and self-reflection as drivers of trainee development	Embedding and fostering intentional reflective practice	EPAs and entrustment discussions supported trainees in regularly self-reflecting on their performance, progress, and learning requirements	“I believe if we there were no EPAs and with the amount of work we have and how busy we are, I don’t think we would get that much time to sit there and self-reflect if the EPAs were not there.” – Intern 12 “It allowed you to track your progress throughout the year based on what was documented. It wasn’t necessarily completely objective, but I could look back and see whether I had stagnated or progressed in certain areas. That helped me to reflect on my development, especially with the more specific tasks.” – Intern 5

	Self-calibration of progress	Entrustment discussions helped trainees improve their self-awareness and focus their developmental goals	“It also helps me, if say, I moved on to a new clinical rotation, and I look back on what I didn’t do so well in a previous rotation, I’m able to work on that, or perhaps reflect on that again and see how I’m tracking.” – Intern 13

	Supervisor feedback as a central mechanism to trainee development	Trainees placed more value on the feedback itself than on the entrustment level assigned to them by their respective supervisors	“I think ultimately, the benefit that I got from the EPA was the word feedback or the written feedback from my preceptor. I would take, I guess, more away from that than the entrustment scale, or where they decided to tick for me.” – Intern 13 “EPAs encouraged discussions with my preceptor that probably wouldn’t have happened otherwise. While there would still have been some dialogue without EPAs, it wouldn’t have been to the same extent as what the EPA process provided.” – Intern 6 “It didn’t matter what level (of entrustment) was achieved, it was the act of self-reflection and feedback that supported learning…” – Intern 3

	Sustaining feedback practice	Trainees received less feedback over time as they progressed through the intern year and were granted greater autonomy in practice	“It reduces the amount of feedback we would be getting as we’re being entrusted more…so there will be no sort of feedback involved in there when you’re doing a task independently, I guess.” – Intern 7

Mismatch between formal entrustment and the trainees’ self-perceived readiness	Self-perceived readiness gaps	Trainees revealed gaps between entrustment and self-perceived readiness, characterised by feeling overwhelmed by expectations and doubts about the credibility of ratings	“Entrustment at level 3 prior to readiness overwhelmed some interns. Once you get level three, you’re on your own, and then that sort of scared me a little bit.” – Intern 4 “It still doesn’t mean I’m confident managing high-risk cases, but I’ve been signed off. From my experience, once pharmacists knew I was fully signed off for reviews, discharges, and histories, they didn’t even want to co-sign anymore — it was like, ‘oh, you’re independent now, you can do it.’” – Intern 9

	Premature and rushed entrustment decisions	Formal entrustment level by supervisor after a certain number of observations not reflecting the trainee’s self-perceived capabilities to manage the variety of cases	“I was signed off on ten level 3 discharge EPAs, but in retrospect they were all simple cases – patients going home with just one or two medicines. Later in my intern year, I started encountering more complex discharges, like Webster packs, nursing homes, hospital transfers, and interstate cases. Technically, I had already been signed off by the EPA, but I was nowhere near competent to manage those kinds of discharges.” – Intern 13

Perceptions and implications upon reaching a finite numerical target leading to autonomy in practice	Transferability of entrustment across different settings and contexts	Misalignment between entrustment level and self-perceived capability	“When I rotated to a ward with more complex patients, I suddenly felt I needed more supervision because the cases were so different. I actually wanted closer support, but it wasn’t provided as readily since I had already been signed off.” – Intern 3 “I think, for example, if you’ve done ten discharge EPAs at level 3, but they’re all for patients going back to the same nursing home – where the requirements are the same and the patients are similar – that’s very different from, say, a clozapine discharge or something more complex. So even if you’ve been signed off in the area of ‘discharges’ overall, it might not really reflect how competent you are across the full range of discharge scenarios.” – Intern 14

	Reality of un-encountered complexities	Transitioning to greater autonomy safely, requires recognition that there will still be uncertainties	“Even once I’m fully registered and practicing as a pharmacist, there will still be complex discharges I’ll need to manage. So the entrustment level alone doesn’t necessarily mean I’m ready for every situation”. – Intern 7 “I don’t know if putting a strict number on it, like saying you must do ten level 3 entrustment observations before you can progress, is the best approach. I think it should be more about doing a few at each level, and then actually monitoring your growth throughout the intern year. Because if you’re not showing growth over time, that’s when you can identify that something isn’t right.” – Intern 11


### Theme 1: Entrustment as a time and task-dependent marker of competence, readiness, and autonomy, with EPAs providing structured learning and goal setting

Interns reported that being entrusted by supervisors bolstered their confidence and affirmed their growing competence, often mirroring the upward trajectory in their self-reported readiness for entrustment levels. Importantly, they conceptualized entrustment not as absolute independence but rather being entrusted to work as an individual within a team context. Interns recognized they could still seek support from supervisors and that they worked as part of a larger team:

*“The very word itself (entrustment) means independent. It doesn’t mean that when you carry out those activities, that you can’t go to your colleagues and ask for their opinion, but once you’re entrusted to do something, that means that you’re the one doing it.” – Intern 1*

Interns noted that entrustment signified more than a procedural requirement; it represented departmental benchmarks of competence and readiness for autonomy. They described how their supervisors’ willingness to grant greater responsibility was closely tied to these benchmarks, with the act of being signed off serving as a marker of both confidence in the intern and practical readiness to work unsupervised:

*“It (entrustment) was a benchmark, and it was used in a practical sense, and not just that it had to be done. Once you were signed off…they (pharmacist) had the confidence you’d be able to do them pretty much independently.” – Intern 2*

Interns recognized EPAs as valuable tools for goal setting and developmental tracking. Moreover, interns highlighted that EPAs supported clear learning objectives and allowed them to track their growth in autonomy throughout the intern year:

*“EPAs were good in creating a plan for us, because they outlined clear tasks that a pharmacist needs to perform and gave us a certain goal to reach them.” – Intern 12*

Interns particularly appreciated that EPA observations were tied to direct supervision, which provided opportunities for real-time learning. Interns consistently highlighted the value of having supervisors present and engaged throughout the training journey. Some expressed concern that without this structure, supervisors may have been less consistently present, highlighting the role of EPAs in ensuring accountability in the learning environment.

### Theme 2: Feedback and self-reflection as drivers of trainee development

Interns consistently emphasized that self-reflection and structured feedback, and not the numerical entrustment level, were most critical to their development. EPA-related conversations prompted deep reflections, helping interns identify their learning needs and recognize their strengths and weaknesses:

*“It forces you to reflect on your own performance and talk about it with your preceptor. You’d reflect on this regardless, but being required to sit down with your preceptor makes you think about it a little more deeply and identify aspects of your performance you could improve on in the future.” – Intern 6*

Interns valued the detailed feedback received from preceptors, particularly in the early stages of the internship, as it helped them understand their practice readiness and establish goals for further development. More importantly, they emphasized the need for high-quality, constructive feedback to support their growth, noting that the quality of feedback did not always meet their expectations, with some actively seeking more critical input from their preceptors:

*“I was quite firm about asking for constructive feedback. My preceptors were always congratulatory if I did something well – which I’m sure most are – but for me, I wanted more than praise. I would ask, ‘What can I do to do this better next time?’” – Intern 4*

However, some interns expressed concern that both the quantity and quality of feedback diminished after reaching level 3 entrustment. They articulated that structured and frequent feedback during entrustment observations were crucial for their progression to level 3. However, once entrusted to work with more autonomy, reduced supervision often meant fewer opportunities for meaningful feedback:

*“Once I’ve gotten to that level 3, there’s sort of reduced feedback, almost. So I felt like it plateaued. I got to this certain level, and obviously there are still people to ask for help, but I guess the feedback wasn’t so hands on as it was at the beginning of the year.” – Intern 11*

### Theme 3: Mismatch between formal entrustment and the trainees’ self-perceived readiness

While interns generally valued EPAs as useful tools for feedback and development, some felt that preceptors did not always use the entrustment process to its fullest potential. In these cases, the focus on completing the minimum required observations to reach level 3 sometimes overshadowed genuine assessment of practice readiness. Some interns described being signed off before they felt fully confident to manage complex clinical work, which contributed to feelings of uncertainty:

*“I’m scared of getting to that (level) 3, because I know that I’m still lacking a lot of things…I feel if I reach the tenth (entrustment at level 3) easily, they will think that I’m competent and confident.” – Intern 10*

In certain settings, some interns expressed doubts about the credibility of high entrustment ratings given by supervisors who were less familiar with EPAs and the entrustment process. This was most evident in the dispensary setting, where interns felt they were entrusted at levels that did not accurately reflect their performance, leading them to question the validity of some entrustment decisions. Rather than focusing on achieving high entrustment ratings, interns valued decisions that aligned with their own perception of practice readiness:

*“Some of the dispensary EPAs I completed were assessed by dispensary pharmacists who had never done clinical work or who came from community pharmacy and hadn’t really used the EPA tool before. They would almost aimlessly mark me off at a level five, which definitely wasn’t representative of my actual performance.” – Intern 3*

Some interns also articulated that the entrustment process could feel rushed and premature, with entrustment to work autonomously not reflecting their own confidence. While many described entrustment as a gradual process, a few suggested that some preceptors’ assessments appeared to be driven by the desire to have the interns working independently:

*“Sometimes I felt entrustment was a little rushed by some preceptors – they just wanted me to practice independently, even though I didn’t always feel ready myself. On paper it looked like I was ready, but personally I wasn’t.” – Intern 7*

### Theme 4: Perceptions and implications upon reaching a finite numerical target leading to autonomy in practice

Interns reflected that achieving the benchmark of ten level 3 entrustment observations for each core EPA – the minimum requirement set by SA Pharmacy to demonstrate readiness for unsupervised practice – did not always capture the variability or complexity of real-world tasks. Some felt the process risked being reduced to a procedural target, with preceptors focusing on meeting the minimum number of observations and achieving level 3 entrustment for the intern, rather than using EPAs as a tool for ongoing development. A few described this approach as reducing entrustment to simply an assessment exercise or numbers game, with implications for how autonomy in practice was conferred:

*“I feel like the scales didn’t really reflect how I progressed over the year, because all we were looking for was one number on the scale.” – Intern 5*

Many interns stressed that summative entrustment should draw from a range of formative experiences involving varied levels of complexity and context. Interns highlighted the importance of a broader variety of entrustment encounters across different tasks and contexts to support genuine readiness for unsupervised practice. While interns did not advocate for setting a strict number of entrustment observations, they emphasized the need for diversity across settings, complexities, and entrustment levels – including beyond level 3 entrustment – to truly capture growth. Importantly, several highlighted that being entrusted in one context should not automatically translate to competence in another:

*“I might have done my first ten (entrustment observations) in a general medicine rotation, but then I’m in oncology for the second half of the year. But because I’ve done ten (entrustment observation), I’m now good for oncology, which is actually not indicative, because I might be good in Gen Med, but not oncology.” – Intern 15*

## Discussion

By examining how intern trainees experience, interpret, and internalize the entrustment process, this study highlights dimensions they viewed as critical to their progression toward unsupervised practice – including structured learning, goal setting, and reflective practice. At the same time, it exposes a central paradox: entrustment, while intended as a developmental tool, may diminish its support for trainees when it is primarily operationalized as an assessment endpoint [31,32,33,54]. Findings from this study further revealed that for some trainees, the focus on meeting numeric benchmarks – particularly meeting the minimum number of level 3 observations for formal sign-off – reinforced perceptions of EPAs as a “numbers game,” [33] where perceived premature entrustment left them feeling uncertain about their practice readiness [31,32,35].

As an assessment *of* learning, EPAs were used as a benchmark of autonomy and readiness for unsupervised practice. As an assessment *for* learning, trainees in this study described how the EPA framework helped them establish structured goals, track their progression, and regularly reflect on their strengths, weaknesses, and growing autonomy throughout the year [31,33]. The EPA framework increased the frequency of direct observations and feedback from supervisors, which helped trainees feel psychologically supported to navigate complex tasks [43,55,56]. Our study demonstrated that trainees viewed entrustment positively when it was experienced as a gradual, progressive reduction in supervision, paired with opportunities for self-reflection and feedback, which in turn enhanced their confidence to manage the complexities of autonomous practice. However, when entrustment is positioned primarily as an assessment *of* learning, it risks overlooking the rich opportunities it holds *for* learning [54]. Our findings reinforce the notion that entrustment should not be seen as an evaluative endpoint but rather as a dynamic and ongoing process [31,32,35]. Even after achieving unsupervised practice, learners continue to benefit from feedback, reflection, and developmental conversations. From the trainees’ perspective, entrustment provided affirmation of competence and a pathway to autonomy in practice, yet at times entrustment also risked being experienced as premature or numbers-driven, leaving some trainees feeling uncertain and underprepared when entrusted at level 3. Viewing autonomy as a simple dichotomy – supervised versus unsupervised – undermines the importance of the developmental journey, where trainees are coached, supported, and progressively entrusted with more responsibility while continuing to receive meaningful feedback and guidance.

Feedback in workplace-based training, when specific and well-timed, can serve as a catalyst for transforming assessment into meaningful developmental learning [57]. In our study, trainees emphasized that verbal feedback from supervisors was far more valuable for their growth than the numeric ratings assigned through entrustment scales, reaffirming findings in previous studies with medical residents [31,32,33]. While entrustment ratings provided a structured representation of supervision levels [34], they offered limited insight into actual progression or readiness, whereas constructive feedback from formative assessments was viewed as central to development. Notably, trainees perceived a decline in the frequency and quality of feedback once they were formally entrusted with level 3 unsupervised practice, which many perceived as premature tapering of support. This perception echoes concerns raised by Ginsburg and colleagues that entrustment ratings may serve more to justify assessment decisions than to promote learning [34]. If the summative function dominates – such as when level 3 is treated as a benchmark to be achieved – the focus on documenting evaluative judgments may overshadow opportunities for formative coaching, potentially contributing to the observed decline in supervisor feedback once trainees were entrusted [34,35,54,58]. While a reduction in feedback is expected as supervision decreases, structured feedback should remain an integral component of early-career trainees’ ongoing development, to ensure continued growth as they assume greater autonomy.

The dissonance between formal entrustment and trainees’ perceived readiness highlights another critical tension in current entrustment frameworks: being deemed ready for unsupervised practice does not necessarily translate into feeling ready [24]. This represents a divergence from the quantitative data, as trainees frequently described being entrusted to work unsupervised before they felt personally ready, despite reporting a median self-perceived readiness for entrustment rating of 3 for most EPAs. Furthermore, some trainees in this study emphasized that the EPA process often felt like a “numbers game,” [33] where meeting the pre-specified minimum required observations to reach level 3 entrustment by a set time point took precedence over genuine assessment of their practice readiness and confidence building. Moreover, reducing entrustment to a minimum number of required observations risks overlooking the contextual variability essential for developing true practice readiness. While there may not be a fixed number of required observations for each task, assessment should prioritize holistic experiences that meaningfully support trainees in their progression toward self-confidence in unsupervised practice [58]. Although some interns articulated feelings of being prematurely entrusted, all 16 successfully completed their licensing examinations and were deemed ready for unsupervised autonomous practice, raising the possibility that self-perceived unreadiness may reflect heightened self-expectations or a lack of confidence, rather than actual gaps in competence [59]. Notably, the interns represented high academic performers within their cohorts, who may be more self-critical and reflective in their evaluations of readiness.

Our empirical findings reinforce Watling and Ginsburg’s argument that entrustment realizes its full developmental potential when assessment is balanced with mentorship and feedback, whereas an overreliance on numerical benchmarks for entrustment decisions risks narrowing its impact [35]. Entrustment decisions that facilitate self-reflection and feedback provide important opportunities to guide, support and personalize training in real time [32,34,35]. By adopting a *coaching*-oriented approach – one that supports learners through ongoing workplace-based interactions – we foster more meaningful learning environments [31,35,58]. Such an approach creates psychological safety, allowing trainees to engage authentically with their developmental needs, explore areas of uncertainty, and strengthen both competence and confidence over time [35,57,60,61]. In these settings, trainees are more likely to internalize feedback, reflect meaningfully on their performance, and feel genuinely prepared to assume the responsibilities of autonomous practice.

Moreover, our study suggests the value of reframing entrustment from a unidirectional process to a more bidirectional one, where trainees are actively engaged in feedback and decision-making conversations. Such an approach has the potential to align perceptions of readiness between supervisors and trainees and ensure that autonomy is granted when both parties feel confident in the trainee’s ability to navigate the complexities of autonomous collaborative practice. Although concerns about overestimation of ability are valid [59], Caro Monroig and colleagues [24] demonstrate how trainees can actively recalibrate supervisor judgments, advocating for levels of supervision that more accurately reflect their developmental stage. A paradigm shift is needed toward models that center the learner within entrustment conversations and view readiness not only as a judgment to be assigned but as a shared, negotiated understanding between trainee and supervisor. In doing so, entrustment becomes more than a threshold to be crossed; it is reframed as a professional journey toward becoming an autonomous, collaborative healthcare practitioner who takes responsibility for clinical decisions, upholds professional standards, and commits to ongoing self-improvement and lifelong learning. Reducing entrustment to numeric values in a unidirectional process risks overlooking autonomy as a core element of professional identity – one that must be nurtured, not just certified [34].

This study has several notable strengths and limitations. The longitudinal collection of intern pharmacists’ self-perceived readiness for entrustment data at three distinct time points, followed by focus group interviews, provided a nuanced understanding of how entrustment is experienced from the trainee’s perspective. However, the absence of the supervisor voice limits the ability to know how trainee-reported experiences align with supervisors’ perceptions. Although some data on the frequency of formative EPA observations were available, these were not consistently documented across rotations and were particularly sparse following summative sign-off at level 3, which limited our ability to examine how ongoing opportunities for observation and feedback influenced changes in interns’ self-perceived readiness for entrustment over time. While the perspectives of the 17 SA Pharmacy interns provide valuable insights, they may not reflect the experiences of all pharmacy interns, particularly those less familiar with EPAs and entrustment principles or with different levels of insight and reflective ability. Broader recruitment across varied contexts and trainee backgrounds would likely have captured a wider spectrum of perspectives and offered a more comprehensive picture of how entrustment is experienced and internalized.

## Conclusion

EPA frameworks and entrustment processes are designed to support trainees in becoming self-directed learners. Our findings suggest that the developmental value of entrustment lay in its coupling with feedback and self-reflection; once level 3 was achieved and trainees began working autonomously, opportunities for ongoing feedback diminished, resulting in potential missed opportunities for learning and growth. A focus on numeric benchmarks risks reducing EPAs to a “numbers game,” overshadowing their intended role in fostering growth, reflection, and professional identity formation. Reframing entrustment as a dynamic, *learner-centered* process grounded in coaching, narrative feedback, and tapered supervision can ensure that EPAs fulfill their potential for cultivating confident, autonomous practitioners.

## Additional File

The additional file for this article can be found as follows:

10.5334/pme.2259.s1 Appendices. Appendix A and B.
