# Supplementary material for: Beyond the Numbers: Exploring Tensions Between Formal Entrustment and Trainee Readiness in Internship Training — A Mixed-Methods Study
Source: Perspect Med Educ. 2026 Mar 19;15(1):279–95. doi: 10.5334/pme.2259 (PMC13004065; doi:10.5334/pme.2259)
Supplement: Appendices. — Appendix A and B. [file pme-15-1-2259-s1.zip › pme-2259_al-diery-s1/Appendix A GRAMMS checklist.docx]

Appendix A: Good Reporting of A Mixed Methods Study (GRAMMS) checklist

| **Guideline** | **Section: page** |
| --- | --- |
| Describe the justification for using a mixed methods approach to the research question | Page 5, lines 114 – 131 |
| Describe the design in terms of the purpose, priority and sequence of methods | Page 5, lines 116 – 120 |
| Describe each method in terms of sampling, data collection and analysis | **Quantitative data:**  Page 6, lines 150 – 171  **Qualitative data:**  Page 7, lines 172 – 218 |
| Describe where integration has occurred, how it has occurred and who has participated in it | Page 9, lines 219 – 231 |
| Describe any limitation of one method associated with the present of the other method | Page 5, lines 121 – 129 |
| Describe any insights gained from mixing or integrating methods | Page 5, lines 121 – 125 |

O'Cathain A, Murphy E, Nicholl J. The quality of mixed methods studies in health services research. J Health Serv Res Policy. 2008;13: 92-98.
