# Supplementary material for: Beyond the Numbers: Exploring Tensions Between Formal Entrustment and Trainee Readiness in Internship Training — A Mixed-Methods Study
Source: Perspect Med Educ. 2026 Mar 19;15(1):279–95. doi: 10.5334/pme.2259 (PMC13004065; doi:10.5334/pme.2259)
Supplement: Appendices. — Appendix A and B. [file pme-15-1-2259-s1.zip › pme-2259_al-diery-s1/Appendix B - SA pharmacy EPA statements.docx]

**Appendix B: Types of SA Pharmacy EPAs**

Each EPA reflects a discrete task performed at discrete points of encounter. For each EPA, the learner is required to reach the defined minimum number of EPAs assessed as achieving a Level 3 Entrustment i.e., able to perform each respective activity independently prior to working independently with reactive supervision. The defined EPA types as well as minimum number requirements and discretions are as outlined in this procedure, refer to applicable sections depending on the category of learner utilising the EPAs and within their scope of practice. With all EPA activities, it is recommended that the learner undertakes the activity in a variety and range of patients and settings i.e., different pre-admission settings (e.g., home, nursing home, interhospital transfers etc), admission settings (e.g., ED, ward etc), varying patient ages, social history, and medication management (e.g., self, carer, dosing aids etc).

The existing list of SA Pharmacy EPAs are as below, the minimum number refers to the number of EPAs assessed as achieving a Level 3 Entrustment:

**Core EPAs (1 – 6)**

1. Dispensing:

1.1 Dispensing inpatient medicines *(minimum 10)*

1.2 Dispensing outpatient medicines *(minimum 10)*

1.3 Dispensing discharge medicines *(minimum 10)*

1.4 Distributing medicines *(minimum 10)*

1.5 Dispensing of Oral Cancer Therapy *(minimum 3, particularly for staff who are likely to regularly dispense oral cancer therapies)*

2. Taking a medication history *(minimum 10)*

3. Undertaking chart annotation, clinical review, and assessment of medication management *(minimum 10)*

4. Undertaking therapeutic drug monitoring *(minimum 10, different varieties)*

5. Facilitating patient discharge or transfer and providing education *(minimum 10)*

6. Providing specific patient education and liaison:

6.1 Assessment of inhaler technique and counselling *(minimum 2)*

6.2 Anticoagulation counselling *(minimum 2 x warfarin, minimum 3 x* *DOACs)*

6.3 Oral Cancer therapy education *(minimum 5 for pharmacists working in dispensary (non-cancer trained) and pharmacists working in oncology*)

**Optional EPAs (7 – 10)**

7. Initiation of clozapine (*minimum 3 for pharmacists working in mental health*)

8. Inter-professional Ward Round *(minimum 10)*

9*.* Regional Cancer services- related tasks:

9.1 Ordering of Cancer Therapies – SLADE sites *(minimum 10)*

9.2 Dispensing of parenteral cancer therapies from an external supplier*(**minimum 10)*

9.3 Receiving cancer therapies – SLADE sites *(minimum 10)*

9.4 Safe transport of Cancer Therapies *(minimum 3)*

10. Partnered Pharmacist Medication Charting/Partnered Pharmacist Medication Prescribing (PPMC/PPMP) *(minimum 10)*
